# Blocking the blocker

**DOI:** 10.7554/eLife.92076

**Published:** 2023-09-12

**Authors:** Qi Dai

**Affiliations:** 1 Department of Molecular Bioscience, The Wenner-Gren Institute, Stockholm University Stockholm Sweden

**Keywords:** lncRNA, insulator, Fub-1, bithorax, Ubx, CTCF, *D. melanogaster*

## Abstract

An unexpected interaction between a long non-coding RNA locus and a genetic insulator called *Fub-1* has an important role in gene regulation during development in *Drosophila*.

**Related research article** Ibragimov A, Bing XY, Shidlovskii Y, Levine M, Georgiev P, Schedl P. 2023. lncRNA read-through regulates the BX-C insulator Fub-1. *eLife* **12**:e84711. doi: 10.7554/eLife.84711.

Genes that code for proteins account for less than 2% of the human genome: what does the rest of the genome do? Some of it is given over to genes for RNA molecules that have vital roles in cellular processes, such as ribosomal RNAs, transfer RNAs and small regulatory RNAs. However, the roles of other RNA molecules – notably molecules called long non-coding RNAs (lncRNAs) – are not fully understood, although many lncRNAs are known to be involved in the regulation of gene transcription (Statello et al., 2021).

The non-coding part of the genome also contains regions called cis-regulatory DNA elements that orchestrate the precise timing, location and extent of gene expression (Oudelaar and Higgs, 2021). These elements include chromatin boundary elements, also known as insulators, that associate with insulator proteins to wire the genomic DNA into specific three-dimensional shapes (Phillips-Cremins and Corces, 2013). Insulators also directly regulate gene expression by separating other regulatory elements, such as enhancers and promoters.

Gene regulation means that the different cell types in a multi-cellular organism decode the same genetic information (the genome) in a cell-type-specific manner. One widely studied example of this is the regulation of hox genes, which have an important role during animal development (Hubert and Wellik, 2023). However, the precise mechanisms governing the spatial and temporal expression of hox genes are still under investigation. Now, in eLife, Airat Ibragimov, Paul Schedl and colleagues at Princeton University and the Russian Academy of Sciences report the results of experiments on *Drosophila* which reveal an unexpected role for lncRNAs in the regulation of hox genes (Ibragimov et al., 2023).

Focusing on the regulation of a hox gene called *Ubx* (which is short for *Ultrabithorax*), the researchers studied the function of *Fub-1*, an insulator that is positioned between the regulatory domains that determine how *Ubx* is expressed in two distinct body segments in *Drosophila* embryos (Figure 1; Castelli-Gair et al., 1992; Maeda and Karch, 2015).

**Figure 1.**
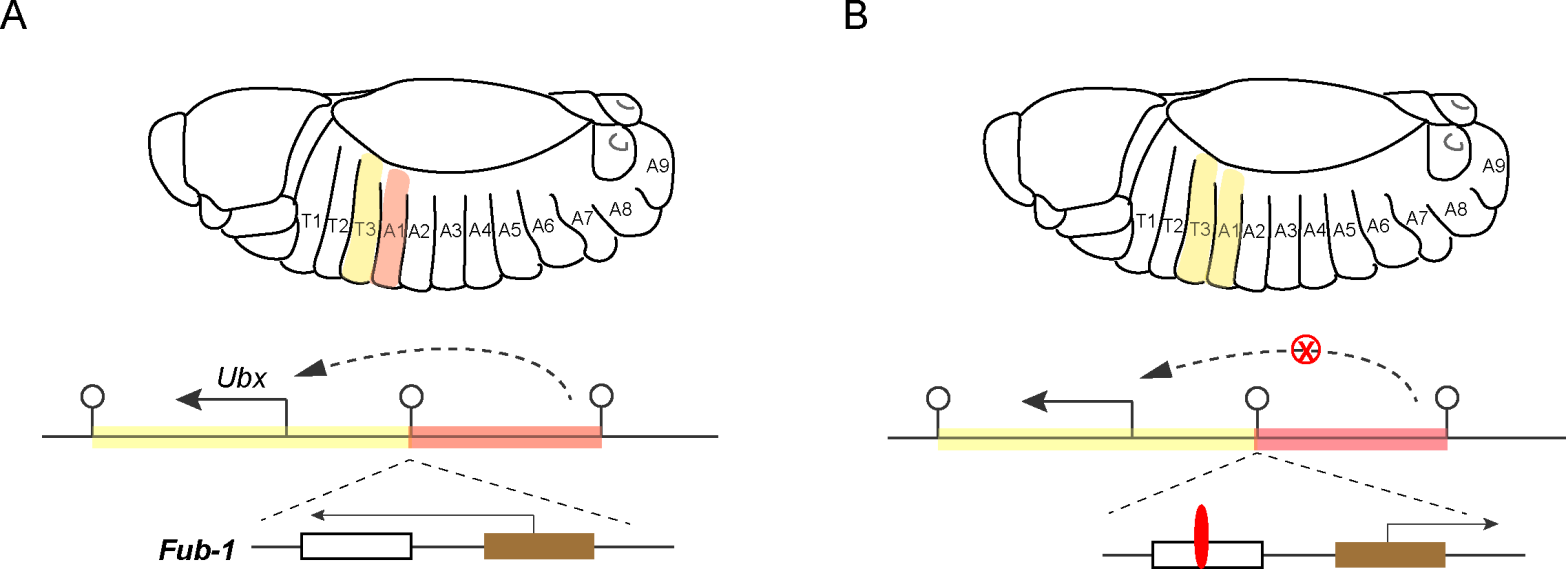
Hox genes, insulators and lncRNAs. (**A**) Schematic drawing of a *Drosophila* embryo showing thoracic and abdominal body segments (top), and the locus of the hox gene called *Ubx* (bottom); the solid line with the arrowhead indicates the direction of *Ubx* transcription. The yellow region is the regulatory domain that is responsible for specifying *Ubx* expression in the T3 body segment, and the orange region is the regulatory domain for the A1 body segment (dashed line with arrowhead). The *Fub-1* insulator (lollipop symbol in the middle) that separates these two domains contains two sub-elements: the *HS1* sub-element (white rectangle) contains a binding site for an insulator protein called CTCF, and the *HS2* sub-element (brown rectangle) contains an element that promotes the transcription of a lncRNA gene (solid line with arrowhead). There is also an insulator at the other end of each regulatory domain. Ibragimov et al. have shown the activity of the *Fub-1* insulator is compromised when the lncRNA in *HS2* is being actively transcribed. (**B**) When the direction of lncRNA transcription is reversed, the interaction between the regulatory domain for A1 (orange region) and the *Ubx* gene is blocked (red X), presumably because the insulator protein CTCF (red) is able to bind to the *HS1* element, which results in the A1 segment resembling the T3 segment.

*Fub-1* has two parts: a sub-element called *HS1* contains a binding site for an insulator protein, and a sub-element called *HS2* promotes the transcription of a lncRNA gene (Pease et al., 2013). Ibragimov et al. employed a state-of-the-art gene replacement strategy to generate mutants in which *Fub-*1 had been deleted, and mutants in which *Fub-1* had been modified. Surprisingly, the former appeared morphologically normal (although there were changes to the three-dimensional shapes adopted by the genomic DNA). This was unusual because depleting a classic insulator element typically results in abnormal segmental identity (Gyurkovics et al., 1990).

Further tests using mutant animals with modified *Fub-1* revealed that transcription of the lncRNA promoter in *HS2* counteracts the insulator activity of *Fub-1*. In cells where the lncRNA is actively being transcribed, this presumably happens because the presence of the molecular machinery required for transcription disrupts the binding of the insulator protein to HS1. This dual nature explains why deleting *Fub-1* did not result in a change of morphology. However, when the direction of transcription is reversed, the insulator activity of *Fub-1* is restored. In this case, the regulatory domain for the A1 body segment can no longer interact with the *Ubx* gene, which leads to defects in segment identity (Figure 1).

One puzzling point remains: why does the deletion of *Fub-1* not result in any obvious phenotype? As discussed by Ibragimov et al., this element is conserved in distantly related *Drosophila* species, suggesting that it may be important in other species or in challenging environments, such as the wild.

While the sequence and location of insulators have been mapped in several genomes, the functions and regulation of these elements await further detailed study. The work of Ibragimov et al. elegantly illustrates the multifaceted role of insulators (and lncRNAs), and the complexity of their function. Moreover, although it was carried out in *Drosophila*, their work has broader implications as the function, regulation and chromosomal clustering of many hox genes are evolutionarily conserved, so similar mechanisms may be at play in other animal species.
